# BDSER-InceptionNet: A Novel Method for Near-Infrared Spectroscopy Model Transfer Based on Deep Learning and Balanced Distribution Adaptation

**DOI:** 10.3390/s25134008

**Published:** 2025-06-27

**Authors:** Jianghai Chen, Jie Ling, Nana Lei, Lingqiao Li

**Affiliations:** School of Computer Science and Information Security, Guilin University of Electronic Technology, Guilin 541004, China; 22032303010@mails.guet.edu.cn (J.C.); 23032307052@mails.guet.edu.cn (J.L.); 23032303016@mails.guet.edu.cn (N.L.)

**Keywords:** near-infrared spectroscopy, model transfer learning, SE, balanced distribution adaptation

## Abstract

Near-Infrared Spectroscopy (NIRS) analysis technology faces numerous challenges in industrial applications. Firstly, the generalization capability of models is significantly affected by instrumental heterogeneity, environmental interference, and sample diversity. Traditional modeling methods exhibit certain limitations in handling these factors, making it difficult to achieve effective adaptation across different scenarios. Specifically, data distribution shifts and mismatches in multi-scale features hinder the transferability of models across different crop varieties or instruments from different manufacturers. As a result, the large amount of previously accumulated NIRS and reference data cannot be effectively utilized in modeling for new instruments or new varieties, thereby limiting improvements in modeling efficiency and prediction accuracy. To address these limitations, this study proposes a novel transfer learning framework integrating multi-scale network architecture with Balanced Distribution Adaptation (BDA) to enhance cross-instrument compatibility. The key contributions include: (1) RX-Inception multi-scale structure: Combines Xception’s depthwise separable convolution with ResNet’s residual connections to strengthen global–local feature coupling. (2) Squeeze-and-Excitation (SE) attention: Dynamically recalibrates spectral band weights to enhance discriminative feature representation. (3) Systematic evaluation of six transfer strategies: Comparative analysis of their impacts on model adaptation performance. Experimental results on open corn and pharmaceutical datasets demonstrate that BDSER-InceptionNet achieves state-of-the-art performance on primary instruments. Notably, the proposed Method 6 successfully enables NIRS model sharing from primary to secondary instruments, effectively mitigating spectral discrepancies and significantly improving transfer efficacy.

## 1. Introduction

In recent years, deep learning techniques have demonstrated significant potential in chemometrics due to their powerful feature extraction and pattern recognition capabilities [1,2]. Particularly in Near-Infrared Spectroscopy (NIRS) analysis, these methods have overcome the limitations of traditional approaches such as Partial Least Squares regression (PLS) and manual feature engineering by enabling end-to-end feature learning, thereby providing innovative solutions for the rapid detection and quantitative analysis of material components [3]. However, spectral data discrepancies across instruments in practical applications restrict model generalizability, prompting researchers to develop model transfer methods to enhance cross-instrument prediction performance. The heterogeneous instrumentation, environmental variability, and sample diversity prevalent in industrial scenarios pose substantial challenges to conventional modeling frameworks. Transitioning from laboratory-grade instruments to field-deployed portable devices introduces mismatches in spectral data caused by differences in optical resolution, detector sensitivity fluctuations, and environmental factors such as temperature and humidity. These factors lead to multi-scale distribution shifts and domain-specific feature space misalignment, resulting in significant performance degradation of pre-trained models in new operational contexts [4,5,6,7,8]. Repeated model recalibration to address these variations requires substantial labeled data and incurs high temporal and economic costs, severely limiting the scalable application of analytical models. To address these challenges, researchers have proposed instrument-specific calibration transfer techniques such as Direct Standardization (DS) and PLS-based calibration transfer. While these methods reduce inter-instrument discrepancies by correcting spectral data or model parameters, they remain limited in handling complex distribution shifts due to insufficient adaptability to nonlinear data patterns and neglect of multi-scale features [9,10,11].

The emergence of transfer learning techniques offers a promising solution to this challenge [12,13]. Unlike conventional modeling approaches that heavily rely on target-domain data, transfer learning mitigates model degradation caused by data distribution discrepancies and feature space heterogeneity through knowledge transfer mechanisms, enabling effective adaptation from source to target domains [14,15]. Recently, transfer learning has gained significant attention in cross-instrument model transfer for NIRS applications. For instance, Mishra et al. [16] proposed a deep calibration transfer method based on transfer learning that operates without standard samples, demonstrating applicability for model transfer between FT-NIR and handheld NIR instruments. Zhang et al. [17] introduced a partial transfer component regression framework for calibrating NIRS data in soil analysis, achieving accurate cross-domain predictions through an automated calibration module. Liang et al. [18] further developed an improved segmented direct standardization approach, where transfer samples selected based on specific attributes can be directly applied to calibration models for other attributes, yielding satisfactory results. Despite these advancements, most studies focus on specific transfer scenarios or single distribution alignment strategies. They fail to adequately address the multi-scale characteristics of spectral data and the compound distribution shifts arising from instrumental differences, leaving critical challenges unresolved in practical implementations.

Existing studies have preliminarily validated the feasibility of transfer learning in NIRS applications. Li et al. developed a three-stage transfer framework based on feature mapping alignment, achieving higher classification accuracy and scalability in multi-variety, multi-manufacturer NIRS classification experiments compared to established methods such as SVM, BP, AE, and ELM [19]. Zhang’s team further proposed a spectral-density correlation transfer model that compensates for moisture content differences through an automated calibration module, achieving optimal density prediction for larch datasets under varying moisture conditions [20]. Deng et al. introduced three transfer learning strategies applied to deep learning models for toxin spectral data, effectively addressing the poor adaptability of single-source models [21]. While these studies demonstrate the potential of transfer learning in NIRS, most focus on specific transfer scenarios or single distribution alignment strategies. They inadequately address the multi-scale characteristics of spectral data and the compound distribution shifts caused by instrumental differences, leaving critical challenges unresolved in practical implementations.

Current research faces two critical challenges: First, the multi-scale characteristics of spectral data require models to dynamically adapt to varying wavelength resolutions. Second, instrument-induced marginal and conditional distribution shifts must be jointly optimized to achieve cross-domain alignment. To address these issues, researchers have explored diverse strategies. In feature extraction, multi-scale convolutional neural networks have been proposed to capture spectral characteristics across varying wavelength resolutions [22]. For distribution alignment, domain adaptation techniques such as Maximum Mean Discrepancy (MMD) and adversarial training have been applied to reduce feature distribution discrepancies between source and target domains [23]. However, these methods often separately optimize feature extraction or distribution alignment, failing to achieve synergistic optimization between these components. Therefore, designing a transfer learning framework that dynamically aligns feature distributions while enhancing cross-domain prediction accuracy has become a critical research focus.

Based on this background, this study proposes a novel transfer learning framework, BDSER-InceptionNet, which innovatively integrates a network architecture with a Balanced Distribution Adaptation (BDA) algorithm to simultaneously optimize feature representation and distribution alignment for addressing cross-instrument model transfer challenges in NIRS. First, a foundational feature extraction module is constructed using depthwise separable convolution [24], decoupling spatial feature learning from channel-wise information aggregation. This design ensures a lightweight architecture while enhancing sensitivity to localized spectral characteristics. Second, an RX-Inception multi-scale structure is developed by combining the depthwise separable convolution advantages of Xception with the residual connection properties of ResNet [25]. Finally, an SE channel attention mechanism [26] is incorporated to dynamically recalibrate channel weights, strengthening the representation of critical spectral bands. These improvements effectively resolve issues of feature redundancy and multi-scale information loss inherent in traditional convolutional neural networks for spectral analysis.

More importantly, this study introduces the “Balanced Distribution Adaptation (BDA) theory” into the field of NIRS quantitative analysis for the first time. In cross-instrument NIRS applications, both marginal and conditional distributions of spectral data may shift due to instrumental discrepancies. In contrast to conventional domain adaptation methods that focus solely on marginal distribution alignment, the BDA algorithm jointly optimizes marginal distribution discrepancies and conditional distribution mismatches by establishing a “dual-constraint dynamic adaptation framework”. This approach comprehensively reduces domain divergence while achieving an optimal balance between inter-domain alignment and intra-domain prediction performance, thereby enhancing cross-domain predictive capabilities.

To further evaluate the experimental effectiveness, this study designs six distinct model transfer strategies to compare and analyze the impact of different transfer approaches on migration performance. The corn and pharmaceutical datasets are selected for experiments, as they represent canonical application scenarios of Near-Infrared Spectroscopy (NIRS) in agricultural and industrial domains, respectively, offering broad sector coverage and practical challenges. The corn dataset involves multi-component quantitative analysis, while the pharmaceutical dataset encompasses complex chemical compositions and stringent quality control requirements—both posing high demands on model accuracy and robustness. These datasets, widely adopted as established benchmarks in model transfer and calibration transfer research, enable rigorous validation of the proposed method’s generalization capability and practical applicability.

The experimental results demonstrate that the BDSER-InceptionNet model achieves high-precision prediction of pharmaceutical and corn composition without requiring complex preprocessing. Through the “BDA-optimized transfer learning strategy”, the model significantly outperforms baseline methods such as PLS, SVR, and traditional CNNs in cross-instrument prediction tasks, highlighting its advantages in handling multi-scale spectral data and cross-domain distribution alignment. This outcome not only validates the academic merit of the proposed approach but also establishes a foundation for its practical application in multi-instrument NIR spectroscopy analysis.

## 2. Materials and Methods

### 2.1. Dataset

In this study, we utilized two publicly available near-infrared spectroscopy datasets: the tablet dataset and the corn dataset. These datasets are widely employed in the fields of chemometrics and machine learning to validate the transfer performance of models across different instruments. The following sections provide a detailed description of the characteristics, measurement methods, and experimental roles of these two datasets.

#### 2.1.1. Pharmaceutical Dataset

**Figure 1:**
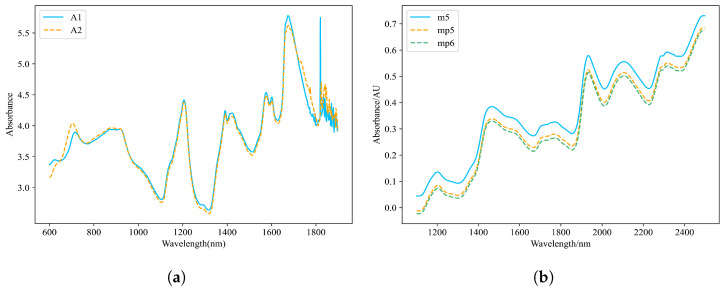
Spectral distribution of the tablet dataset and corn dataset. (**a**) Comparison of spectral profiles between instruments A1 and A2. (**b**) Comparison of spectral profiles between the host instrument (m5) and client instruments (mp5 and mp6).

The dataset consists of 655 tablet samples, with quality, hardness, and active ingredient content serving as the target variables for prediction. Each sample was measured using two near-infrared spectrometers (A1 and A2), resulting in a total of 1310 spectral datasets. The spectral wavelength range spans from 600 nm to 1898 nm, recorded at intervals of 2 nm, covering 650 discrete wavelength points. Despite hardware configuration, environmental conditions, and calibration parameter differences between the host instrument (A1) and slave instrument (A2), their spectral response curves exhibit high similarity in the pharmaceutical dataset, as illustrated in Figure 1a. The dataset is available for download at https://eigenvector.com/resources/data-sets/#idrc2002 (accessed on 10 December 2024).

#### 2.1.2. Corn Dataset

The corn dataset consists of 80 samples, with target components including moisture, oil, protein, and starch content. Each sample was measured using three near-infrared spectrometers (m5, mp5, and mp6), resulting in a total of 240 spectral datasets. The spectral wavelength range spans from 1100 nm to 2498 nm, recorded at intervals of 2 nm, covering 700 discrete wavelength points. Notably, in the corn dataset, significant spectral discrepancies exist between the host instrument (m5) and slave instruments (mp5 and mp6), as illustrated in Figure 1b, which may stem from inconsistencies in hardware configuration, environmental conditions, or calibration parameters. This characteristic poses challenges for cross-instrument model transfer. The dataset is available for download at https://eigenvector.com/resources/data-sets/#corn-sec (accessed on 10 December 2024).

### 2.2. Network Architecture

#### 2.2.1. DSC Structure

Depthwise Separable Convolution (DSC) is an efficient convolutional operation comprising two main components: Depthwise Convolution (DWConv) and Pointwise Convolution (PWConv). Compared to traditional convolutional neural networks, DSC exhibits superior computational efficiency and a smaller model size, leading to outstanding performance across various application scenarios, including image classification [27], object detection [28], and semantic segmentation [29]. Depthwise Convolution is responsible for extracting spatial features from the input data by independently applying convolution kernels to each channel. This approach not only captures spatial information within the data but also significantly reduces computational complexity. Subsequently, Pointwise Convolution aggregates the outputs from all channels using 1 × 1 convolution kernels, integrating information across different channels to produce the final output features. Through this mechanism, DSC enhances model performance while reducing the number of parameters, thereby mitigating the risk of overfitting. This makes it particularly well-suited for scenarios with small sample sizes.

#### 2.2.2. RX-Inception Structure

**Figure 2:**
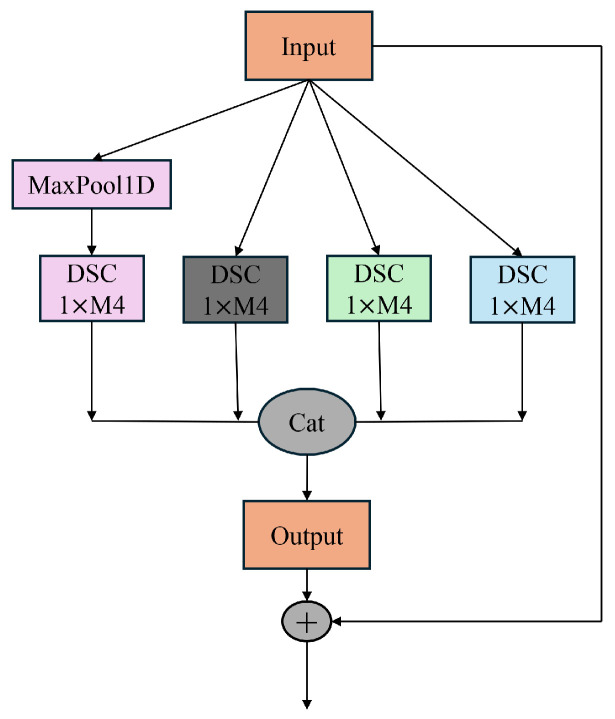
Structure of RX-inception.

Different convolution kernels can lead to variations in the extracted spectral information, resulting in the loss of critical spectral details and consequently reducing efficiency. Spectral data inherently contain multi-scale information, and the differences between spectra obtained from various spectrometers are also multi-scale in nature. Therefore, to address the characteristics of near-infrared spectral data, a multi-scale network was developed. This network incorporates a convolution downsampling strategy tailored to different resolutions, constructing an adaptive input structure for spectral data to mitigate the performance degradation caused by the multi-scale nature of the data. The proposed approach innovatively enhances the Xception architecture and integrates the ResNet structure, deepening the network layers to enable the extraction of richer feature hierarchies. Additionally, this design effectively mitigates the issue of vanishing gradients in deeper networks. The structure of the proposed model is illustrated in Figure 2.

#### 2.2.3. SE Structure

**Figure 3:**
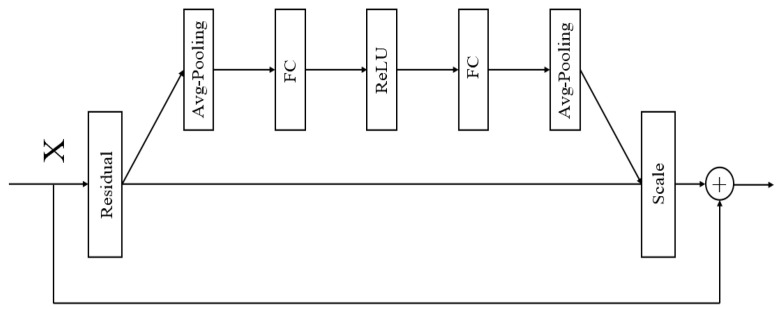
Structure of SE.

The Squeeze-and-Excitation (SE) module is an attention mechanism within neural network architectures.It employs a “Squeeze” operation to capture global information and an “Excitation” operation to assign distinct weights to each channel, thereby recalibrating the channel-wise feature weights. The primary objective of the SE module is to enhance the network’s selective focus on features by learning the importance of each channel, thus improving the network’s representational capacity. In models such as convolutional neural networks (CNNs), feature maps across different channels may contain varying degrees of significant information. The channel-wise attention mechanism allocates different weights to each channel, amplifying the network’s focus on critical features while diminishing the influence of less relevant ones. This approach enables the network to utilize feature information more effectively, thereby enhancing the overall performance and robustness of the model. The structure of the SE module is illustrated in Figure 3.

The SE module recalibrates the weights of feature channels through a three-step process. First, the SE module performs Global Average Pooling, compressing the feature map of each channel into a single scalar value to capture global spatial information. Next, it generates channel-specific weights using two fully connected layers followed by activation functions (ReLU and Sigmoid) [30,31]. Finally, the SE module multiplies these weights with the original convolutional output feature maps, achieving a weighted enhancement of the feature maps and enabling the network to focus more effectively on information from critical channels.

#### 2.2.4. BDSER-InceptionNet Structure

**Figure 4:**
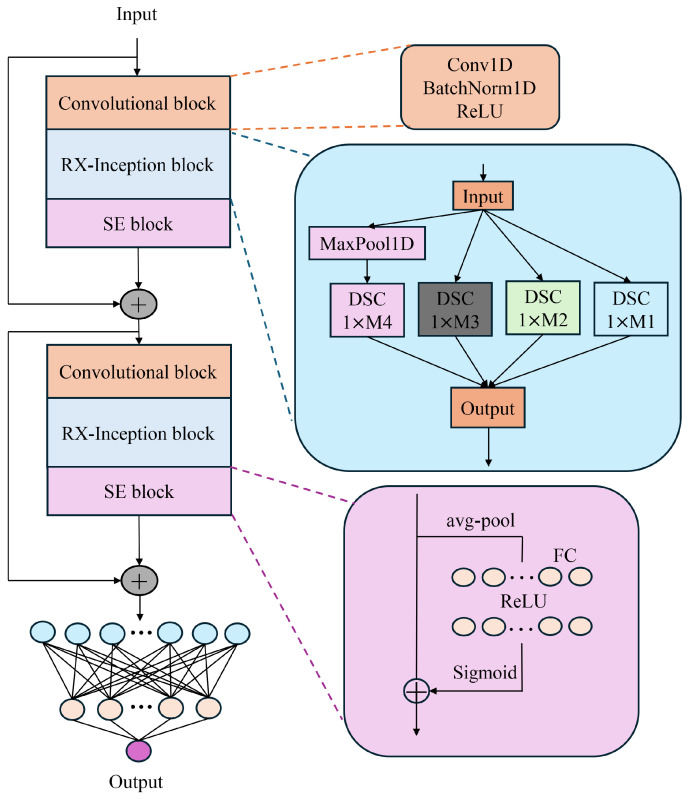
Structure of BDSER-InceptionNet.

As illustrated in Figure 4, the BDSER-InceptionNet model has been meticulously designed and optimized to address the complex characteristics of near-infrared spectral data. The model first employs convolutional downsampling techniques to construct a multi-scale spectral fusion module, effectively enabling the input of multi-scale spectral data and adeptly resolving the issue of dimensional discrepancies in spectral data. Subsequently, by incorporating parallel feature extraction modules, the model can simultaneously capture both local spectral features and global feature dependencies, laying a solid foundation for subsequent analysis. Following the initial convolutional operations, the model integrates the Xception and ResNet architectures and incorporates the Squeeze-and-Excitation (SE) module. By multiplying the weights generated by the SE module with the original convolutional feature maps, this design achieves a weighted enhancement of the feature maps, significantly improving the network’s focus on critical channel information and thereby enhancing the model’s sensitivity and accuracy in identifying important features. Furthermore, to reduce the number of network parameters, accommodate spectral data across different wavelengths, and mitigate the risk of overfitting, an average adaptive pooling layer is introduced.

Through this series of optimized structures, the BDSER-InceptionNet model demonstrates exceptional performance in processing complex near-infrared spectral data, efficiently extracting and utilizing key features and providing a reliable and effective solution for spectral analysis tasks.

### 2.3. Transfer Methods

#### 2.3.1. Balanced Distribution Adaptation Algorithm

This study proposes a method based on Balanced Distribution Adaptation (BDA) for transfer learning in quantitative tasks. This approach simultaneously minimizes the marginal and conditional distribution discrepancies between the source and target domains, achieving cross-domain feature alignment and thereby enhancing the model’s performance in the target domain. Specifically, BDA is integrated into the total loss function of the pre-trained model. During the retraining process of the pre-trained model, certain network layers continuously compute the transfer loss and adjust parameters in the direction that reduces the overall loss. This process ensures maximum similarity between the features extracted from the source and target domains, facilitating effective model transfer from the master to the slave instrument and significantly improving the model’s cross-domain adaptability and prediction accuracy.

In the transfer learning framework, distribution discrepancies primarily arise from two aspects: marginal distribution discrepancy and conditional distribution discrepancy. The innovation of the BDA method lies in its simultaneous consideration of these two dimensions of distribution discrepancies, achieving optimal feature alignment through an adaptive balancing mechanism.

Marginal Distribution Adaptation [32] focuses on aligning the overall feature distributions of the source and target domains. It employs Maximum Mean Discrepancy (MMD) to quantify the distribution differences between the two domains in the feature space. By calculating the MMD value between the source and target domain features, their similarity is assessed, enabling effective alignment of the feature distributions across the two domains.

(1) $$Loss_{\mathrm{MDA}}=\left(X_{s},X_{t}\right)$$

(2) $$Loss_{\mathrm{MMD}}={∥\frac{1}{n}\sum_{i=1}^{n}\Phi \left(X_{s_{i}}\right)-\frac{1}{m}\sum_{i=1}^{m}\Phi \left(X_{t_{i}}\right)∥}_{H}$$

In Equation (2), $X_{s_{i}}$ and $X_{t_{i}}$ represent the features of the source domain and the target domain, respectively. ${∥\cdot ∥}_{H}$ represents the norm in Hilbert space H, which is used to compute the distance between two mapped distributions.

Conditional Distribution Adaptation [33] focuses on aligning the distribution characteristics of the source and target domains at the model output level. In regression task scenarios, where the model outputs continuous values, the Maximum Mean Discrepancy (MMD) between the source and target domain outputs can be computed to quantify the extent of their conditional distribution differences. This approach effectively captures the discrepancies in the distribution of predicted values between the source and target domains, thereby enhancing the model’s generalization ability and ensuring more robust and consistent prediction performance across different domains.

(3) $$Loss_{\mathrm{CDA}}=\mathrm{MMD}\left(Y_{s},Y_{t}\right)$$

In Equation (3), $Y_{s}$ and $Y_{t}$ represent the model outputs of the source domain and the target domain, respectively.

Balanced Distribution Adaptation (BDA) integrates the marginal distribution discrepancy and conditional distribution discrepancy to construct a comprehensive loss function, termed BDALoss. A balancing parameter $\lambda_{1}$ is introduced in this loss function to adjust the weight ratio between the two types of discrepancies. In practical applications, this balancing parameter can be flexibly tuned according to the characteristics and requirements of different tasks, enabling precise control over the influence of marginal and conditional distribution discrepancies.

(4) $${Loss}_{\mathrm{BDA}}={Loss}_{\mathrm{MDA}}+\lambda_{1}{Loss}_{\mathrm{CDA}}$$

This study focuses on the quantitative analysis of near-infrared spectroscopy models, which is inherently a regression task. Therefore, the Mean Squared Error (MSE) loss function was adopted as the task loss function. The mathematical expression of this loss function is as follows:

(5) $${Loss}_{\mathrm{task}}={Loss}_{\mathrm{MSE}}=\frac{1}{n}\sum_{i=1}^{n}{\left(y_{i}-\hat{y_{i}}\right)}^{2}$$

In Equation (5), $y_{i}$ represents the true value, ${\hat{y}}_{i}$ denotes the predicted value, and the total loss function for the model during retraining is:

(6) $${Loss}_{\mathrm{total}}={Loss}_{\mathrm{task}}+\lambda_{1}{Loss}_{\mathrm{BDA}}$$

(7) $${Loss}_{\mathrm{total}}={Loss}_{\mathrm{MSE}}+\lambda_{1}{Loss}_{\mathrm{MDA}}+\lambda_{1}\lambda_{2}{Loss}_{\mathrm{CDA}}$$

**Figure 5:**
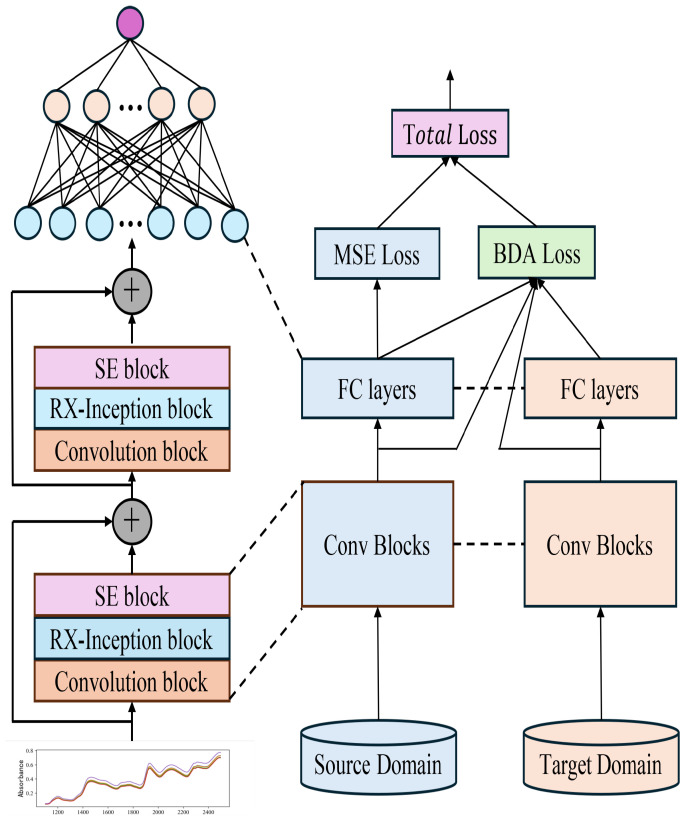
Schematic diagram of the model transfer method incorporating BDA.

The total loss function is given by Equations (6) and (7), where $\lambda_{1}$ and $\lambda_{2}$ are used to adjust the weights of the BDA loss and CDA loss, respectively, to balance their contributions to the total loss. The task-specific loss function, ${\mathrm{Loss}}_{\mathrm{task}}$, is determined based on the type of task. For instance, in regression tasks, the Mean Squared Error (MSE) loss function is typically employed, whereas in classification tasks, the cross-entropy loss function can be used. This is illustrated in detail in Figure 5.

#### 2.3.2. Model Transfer Strategy

**Figure 6:**
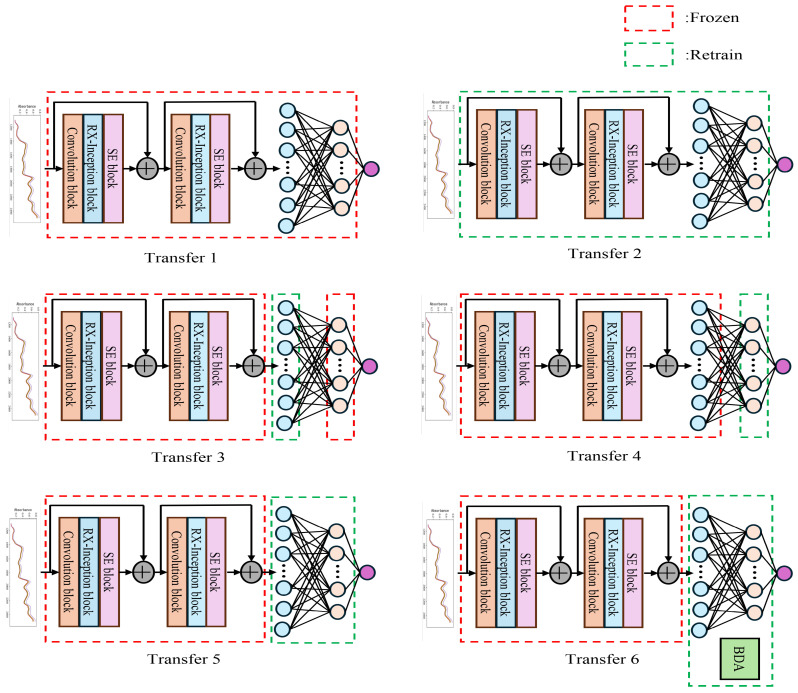
Six different model transfer methods.

The motivation for freezing certain layers mainly lies in balancing the preservation of general features, adapting to domain-specific characteristics and conserving computational resources. Convolutional Neural Network (CNN) layers typically capture low-level and mid-level features that are general and transferable across different domains or devices. Freezing these layers helps preserves the learned robust features, reduces the risk of overfitting to the target domain data, and saves computational resources. Fully connected layers capture high-level representations and decision boundaries that are often more domain-specific. Fine-tuning these layers allows the model to adapt to the unique characteristics of the target domain, such as differences in data distribution or feature sensitivity between devices. By freezing part of the network, the number of parameters that need to be retrained is reduced, leading to faster transfer learning and lower computational demands. Therefore, this study designs the following six model transfer strategies to investigate how different network architectures affect transfer performance under various transfer settings. The detailed configuration is illustrated in Figure 6.

- Transfer 1: Freeze all layers without updating parameters and directly perform testing. The primary motivation is to evaluate the baseline performance of the pre-trained model on the target device without any adaptation. This approach assumes that the features and weights learned from the source device are directly transferable, relying on the model’s generalization ability. By freezing all layers, no retraining is required, minimizing computational overhead and making this method resource-friendly. This is particularly useful when computational resources are limited or when a quick evaluation is needed.
- Transfer 2: Transfer all layers and parameters of the network trained on the host instrument without freezing any layers, allowing the entire network to be fine-tuned and updated.The motivation is to fully adapt the model to the target domain by updating all parameters. This is ideal when the source and target devices differ significantly in data distribution or feature sensitivity, requiring adjustments to both low-level features (convolutional layers) and high-level representations (fully connected layers). Fine-tuning the entire network provides maximum flexibility, enabling the model to learn device-specific patterns at all levels of abstraction, from general features to decision boundaries. However, This method demands substantial computational resources and a sufficient amount of labeled data on the target device. There is also a risk of overfitting if the target dataset is small, but it offers the greatest potential for performance improvement when domain differences are pronounced.
- Transfer 3: Fix the convolutional layers and the second fully connected layer, and retrain the first fully connected layer. Freezing the convolutional layers retains the low-level and mid-level feature extraction capabilities learned from the source device. These features are assumed to be generalizable across devices, reducing the need for retraining and preventing overfitting to the target domain. Freezing the second fully connected layer preserves the final decision-making process from the source domain. Fine-tuning the first fully connected layer allows the model to adjust intermediate high-level representations to the target domain. This provides a compromise between preserving general knowledge and enabling some adaptation, targeting the layer where domain-specific differences may first emerge. By limiting retraining to the first fully connected layer, this method reduces computational complexity compared to full fine-tuning while still allowing partial adaptation.
- Transfer 4: Fix the convolutional layers and the first fully connected layer and retrain the second fully connected layer. Freezing the convolutional layers maintains the general feature extraction capabilities, consistent with the assumption that these features are robust across devices. Freezing the first fully connected layer preserves the intermediate high-level representations learned from the source domain, suggesting that these are sufficiently transferable to the target device. Fine-tuning the second fully connected layer focuses on adapting the final perdictions process to the target domain, allowing the model to adjust its output predictions.
- Transfer 5: Fix the convolutional layers and retrain both the first and second fully connected layers. Freezing the convolutional layers retains the robust, transferable features extracted from the source domain, avoiding unnecessary retraining of these layers. Fine-tuning both the first fully connected layer and the second fully connected layer allows the model to adjust both the intermediate high-level representations and the final decision-making process to the target domain. This approach is motivated by the need to adapt the network’s higher-level processing to device-specific characteristics. This method strikes a balance between computational efficiency (by freezing the convolutional layers, which typically contain the majority of parameters) and adaptability (by retraining the fully connected layers). It is effective when the differences between devices are concentrated in high-level representations.
- Transfer 6: Fix the convolutional layers, retrain both the first and second fully connected layers and introduce BDA during retraining, combining it with the mean squared error loss function to form the total loss. Freezing the convolutional layers maintains the general feature extraction capabilities consistent with Methods 3–5 under the assumption that these features are transferable across devices. Fine-tuning the first fully connected layer and the second fully connected layer with BDA is motivated by the need to explicitly address domain shift. BDA balances the marginal and conditional distributions between the source and target domains, reducing feature distribution discrepancies and improving transferability. Combining BDA with MSE loss ensures that the model minimizes prediction errors (via MSE) while aligning feature distributions (via BDA). This dual-objective approach enhances robustness when significant domain differences exist between devices.

## 3. Model Evaluation

As shown in Equations (8)–(10), the model evaluation metrics include the Coefficient of Determination ($R^{2}$), the Root Mean Square Error (RMSE), and the Mean Absolute Error (MAE). The MAE and RMSE measure the difference between predicted values and true values, while $R^{2}$ is used to evaluate the goodness of fit of the regression model.

(8) $$R^{2}=1-\frac{\sum_{i=1}^{n}{\left({\hat{y}}_{i}-y_{i}\right)}^{2}}{\sum_{i=1}^{n}{\left(\bar{y}-y_{i}\right)}^{2}}$$

(9) $$\mathrm{RMSE}=\sqrt{\frac{1}{n}\sum_{i=1}^{n}{\left({\hat{y}}_{i}-y_{i}\right)}^{2}}$$

(10) $$\mathrm{MAE}=\frac{1}{n}\sum_{i=1}^{n}\left|{\hat{y}}_{i}-y_{i}\right|$$

where $y_{i}$ represents the true value of the *i*-th sample, ${\hat{y}}_{i}$ denotes the predicted value of the *i*-th sample, and $\bar{y}$ represents the mean value of all samples.

## 4. Experimental Preparation and Design

### 4.1. Experimental Preparation

All experiments in this study were implemented using Python 3.9. The Adam optimizer was employed for training deep learning models. For the classical machine learning methods, Support Vector Regression (SVR) and Partial Least Squares (PLS) were implemented using the Scikit-learn library. In the construction of the PLS model, the minimum number of components retained was set to 5. For the SVR model, a linear kernel was selected, and the kernel parameter gamma was empirically determined as 0.001 after extensive experimentation and tuning, in order to achieve optimal model performance. The CNN model used for comparison is identical to the proposed architecture, except that the RX-Inception module was replaced with a standard convolutional layer; all other parameters remained the same. This design allows for a fair comparison to validate the effectiveness of the RX-Inception structure. All deep learning models proposed in this paper are built on the PyTorch framework, with the specific hyperparameters detailed in Table 1.

### 4.2. Experimental Design

The experimental design of this study is as follows: First, outlier sample screening was performed. For the tablet dataset, the leave-one-out cross-validation method was applied, and 19 outlier samples (MSE range: 30.62–795.99) were removed using a mean squared error threshold of 30. The model was constructed based on the first 530 wavelength points and truncated according to the spectral backend noise characteristics. For the maize dataset, due to the limited sample size (n = 80) and the absence of outliers, the full dataset was retained. Second, through comparative analysis, it was determined that no spectral preprocessing was implemented. It was found that the maize dataset achieved optimal $R^{2}$ performance (0.97) without preprocessing, while the tablet dataset showed insignificant improvements after SNV and MSC preprocessing ($\Delta R^{2}$ < 0.02). Regarding sample partitioning strategies, after comparing K-S [34], SPXY [35], and train_test_split algorithms, the K-S algorithm was ultimately selected to enhance model prediction accuracy. During the model training phase, differentiated parameters were used for the primary instrument: batch_size = 128 and epochs = 300 for the tablet dataset, and batch_size = 8 and epochs = 1000 for the maize dataset. Both datasets were configured with the Adam optimizer (initial learning rate of 0.001) and implemented a dynamic learning rate decay strategy (halving the learning rate if no improvement was observed for 30 epochs). When transferring to the secondary instrument, the original training epochs and optimizer settings were maintained, and parameter consistency strategies were employed to enhance model adaptability.

## 5. Results and Discussion

### 5.1. Results of the Pharmaceutical Dataset

Using A1 as the host instrument and A2 as the slave instrument, the initial tablet dataset comprised 155 calibration samples, 40 validation samples, and 460 test samples. To optimize model performance, these three subsets were integrated and re-divided into training and testing sets at an 8:2 ratio using the K-S algorithm. After removing 19 outlier samples, a final set of 509 training samples and 127 testing samples was obtained. A model was established using the training set from A1 and then tested on both A1 and A2. Finally, models were built using PLS, SVR, CNN, and BDSER-InceptionNet for comparison. The results are shown in Table 2.

The data in Table 2 demonstrate that convolutional networks outperform classical chemometric methods in feature extraction. The improved BDSER-InceptionNet model proposed in this paper further enhances predictive performance by incorporating multi-scale fusion, residual structures, and SE attention mechanisms, achieving the best results with an RMSE as low as 2.0243 and an R^2^ as high as 0.9810. Previous studies [36,37] aim to address model adaptability in Near-Infrared (NIR) spectroscopy applications, but they typically rely on traditional chemometric approaches such as Multiplicative Scatter Correction (MSCA) or linear regression-based model updates, focusing primarily on spectral standardization or optimization of linear model parameters. In contrast, our approach leverages deep learning combined with advanced domain adaptation theory (Balanced Distribution Adaptation, BDA) to offer a more comprehensive and automated solution. This highlights the unique advantages of our method in handling multi-scale distribution shifts and cross-domain feature space mismatches caused by instrumental heterogeneity, environmental interference, and sample diversity.

Following the six transfer methods designed in Section 2.3.2, a comparative analysis of the BDSER-InceptionNet model transfer performance was conducted. The training set of A2 data was used for fine-tuning, and the test set was evaluated. The evaluation metrics remained R^2^, RMSE, and MAE. The results are shown in Table 3.

**Figure 7:**
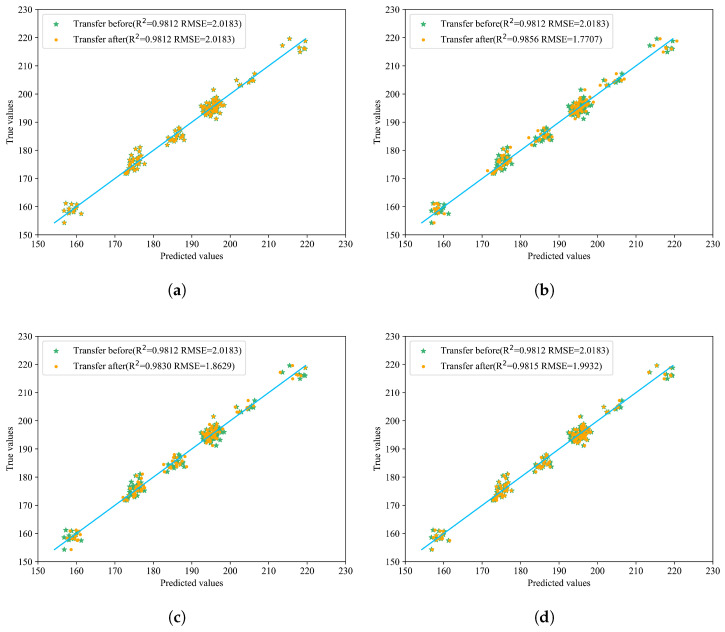

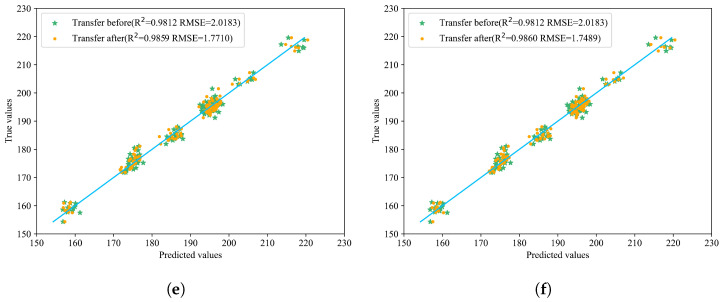
Prediction results of the six model transfer methods. (**a**) Transfer 1: Distribution of predicted and actual values before and after direct prediction with all layers frozen. (**b**) Transfer 2: Distribution of predicted and actual values before and after updating parameters with full participation in transfer learning. (**c**) Transfer 3: Distribution of predicted and actual values before and after transferring only the first fully connected layer. (**d**) Transfer 4: Distribution of predicted and actual values before and after transferring only the second fully connected layer. (**e**) Transfer 5: Distribution of predicted and actual values before and after updating only the last two fully connected layers. (**f**) Transfer 6: Distribution of predicted and actual values before and after transfer learning on the last two layers with BDA.

Compared with the model before transfer, different transfer strategies of various network structures can improve the prediction results. The best performance was achieved after incorporating BDA, with an R^2^ of 0.9860, and RMSE and MAE as low as 1.7489 and 1.4055, respectively, which proves the effectiveness of introducing BDA. The relevant prediction results are shown in Figure 7, which more intuitively displays the effects before and after transfer. In the figure, the yellow points represent the predicted values after model transfer, green points represent the values before model transfer, and the blue line indicates the true values. The closer the prediction points are to the blue line, the better the model’s prediction performance.

Observations from Table 3 and Figure 7 reveal that transferring the entire fully connected layer yields superior overall performance. When employing direct prediction or fine-tuning only the second fully connected layer, most predicted points are closer to the true values; however, certain samples exhibit significant deviations, leading to suboptimal RMSE and R^2^ metrics compared to other transfer methods. Specifically, both R^2^ and RMSE values under these approaches are inferior to those achieved by other transfer strategies. Comparing Transfer 1 (direct testing) with Transfer 2 (full model parameter updates), it is evident that updating the entire model on the new dataset outperforms direct testing. Similarly, Transfer 3 (fine-tuning the first fully connected layer) demonstrates better performance than Transfer 4 (fine-tuning the second fully connected layer). Transfer 5 and Transfer 6 serve as complementary ablation experiments: while Transfer 5 achieves commendable results, Transfer 6 significantly surpasses it in terms of RMSE and MAE improvements. These findings underscore the efficacy of incorporating BDA, which substantially enhances overall performance, thereby validating its practical utility.

### 5.2. Results of the Corn Dataset

In the corn dataset, the data were collected from 80 samples measured by three near-infrared spectrometers: m5, mp5, and mp6. Due to the relatively small sample size, no significant outliers were identified in the dataset, so no preprocessing was performed. As with the pharmaceutical dataset, the three datasets were integrated and re-divided into training and testing sets at an 8:2 ratio using the K-S algorithm, resulting in 64 training samples and 16 testing samples. The m5 instrument was designated as the primary instrument, while mp5 and mp6 served as secondary instruments. The model established on m5 was used to test the datasets of mp5 and mp6. Finally, models based on PLS, SVR, CNN, and BDSER-InceptionNet were developed and compared to predict the four components of maize.

In the prediction experiments for corn moisture, oil, protein, and starch content, the model trained on m5 was directly tested on mp5 and mp6. R^2^ and RMSE were selected as evaluation metrics, and the results are shown in Table 4.

### 5.3. Model Transfer

Using the designed 6 migration methods for BDSER—InceptionNet model transfer, the KS algorithm was applied to divide the mp5 and mp6 datasets into training and testing sets in an 8:2 ratio. Then, the pre-trained model obtained on m5 was fine-tuned on the training sets of mp5 and mp6. The testing was conducted on the corresponding testing sets, with R^2^ and RMSE still chosen as evaluation metrics. Tests were carried out for four components: moisture, oil, protein, and starch. The results are presented in Table 5.

In the experiment of corn moisture content prediction, method 6 achieved the best performance. When transferring the model from the source to the mp5 and mp6 targets, it obtained an R^2^ of 0.8589 and an RMSE of 0.1245 on mp5, and the highest R^2^ of 0.8226 and lowest RMSE of 0.1393 on mp6. These results show a significant improvement in prediction accuracy over method 5, thus proving the effectiveness of BDA. Meanwhile, transferring only the first fully connected layer achieved the second-highest R^2^.

In the corn oil content prediction experiment, when transferring the model from the source to the mp5 and mp6 targets, method 6 achieved an R^2^ of 0.8482 and an RMSE of 0.0636 on mp5. However, on mp6, it was outperformed by method 2, which updated all model parameters. Nonetheless, method 6 still yielded satisfactory results. In contrast, updating only the last fully connected layer consistently achieved the worst outcomes in both model transfers to mp5 and mp6.

In corn protein content prediction, when transferring the model from the source to the m5 target, method 6 achieved the highest R^2^ of 0.9688 and the lowest RMSE of 0.0858. When transferring to the mp6 target, method 6, despite not matching the direct model prediction, still outperformed the other five methods with an R^2^ of 0.9572 and an RMSE of 0.0985, highlighting the effectiveness of BDA in enhancing model transfer.

In the experiment on corn starch content prediction, when the host m5 transfers the model to mp5 and mp6, method 6 achieved an R^2^ of 0.9141 and an RMSE of 0.02123 on mp5, along with an R^2^ of 0.9554 and an RMSE of 0.1478 on mp6. Thus, method 6 performed best in transferring to mp5 and second best in transferring to mp6.

**Figure 8:**
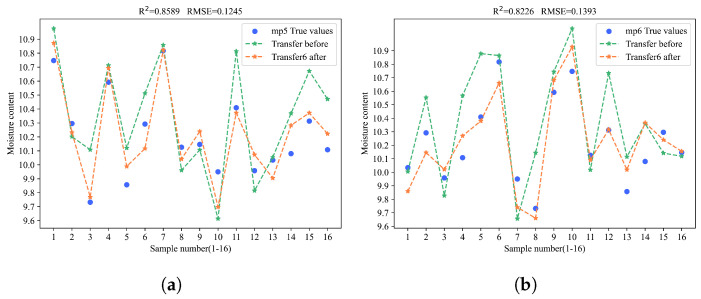

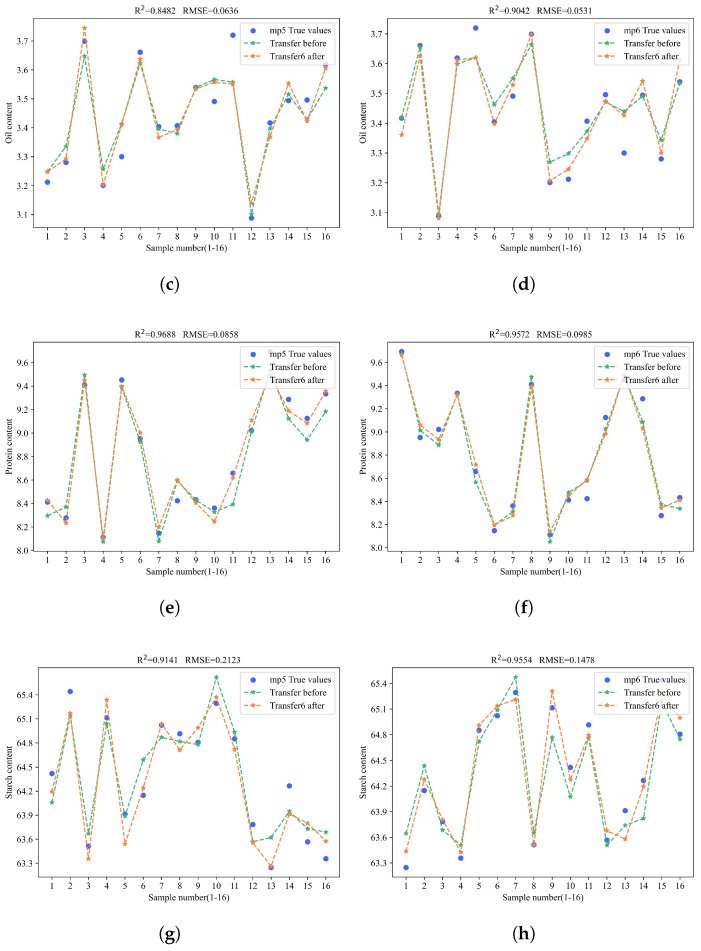
Prediction performance of method 6 for the contents of four substances on mp5 and mp6. (**a**) The prediction performance of method 6 when transferring the model to predict corn moisture content on mp5. (**b**) The prediction performance of method 6 when transferring the model to predict corn moisture content on mp6. (**c**) The prediction performance of method 6 when transferring the model to predict corn oil content on mp5. (**d**) The prediction performance of method 6 when transferring the model to predict corn oil content on mp6. (**e**) The prediction performance of method 6 when transferring the model to predict corn protein content on mp5. (**f**) The prediction performance of method 6 when transferring the model to predict corn protein content on mp6. (**g**) The prediction performance of method 6 when transferring the model to predict corn starch content in mp5. (**h**) The prediction performance of method 6 when transferring the model to predict corn starch content in mp5.

To gain a more intuitive understanding of the model transfer effects, the prediction results of the samples were visualized by plotting the model fitting curves before and after the model transfer. The yellow points indicate the predicted values after the model transfer, the green points indicate the predicted values before the model transfer, and the blue points represent the actual values. The closer the predicted points are to the blue points, the better the prediction performance of the model. As shown in Figure 8.

### 5.4. Comparison Experiment

Comparing BDSER-InceptionNet with PLS/SVR/CNN is not enough to show transfer learning’s advantages. Widely-used model-transfer algorithms include Direct Standardization (DS) [38], Piecewise Direct Standardization (PDS) [39], and Slope and Bias Correction (SBC) [40]. To strictly validate our method’s advantages, we included traditional chemometric methods in the comparison. We built a model on the master instrument using PLS and applied traditional transfer methods. For both the pharmaceutical and maize datasets, we conducted a comparative analysis of component transfer from master to slave instruments. The R^2^ results are in Table 6 and the RMSE results in Table 7.

Through multiple experiments, it has been observed that applying the traditional transfer method, Direct Standardization (DS), as a secondary calibration step can enhance model transfer performance. Experiments conducted on two publicly available datasets—one for corn and one for pharmaceuticals—demonstrate that the proposed BDA algorithm is both efficient and stable, whereas traditional model transfer approaches exhibit significant performance fluctuations. In terms of the $R^{2}$ metric, the DS algorithm performs well on the corn dataset but leads to over-correction and poor results on the pharmaceutical dataset. The PDS algorithm achieves an $R^{2}$ of 0.9556 on the pharmaceutical dataset, outperforming DS and SBC. However, it yields negative $R^{2}$ values on the corn dataset. In contrast, the proposed Transfer 6 method demonstrates superior performance in model transfer. By incorporating a transfer loss during the fine-tuning of the pre-trained model, Transfer 6 consistently improves prediction accuracy and stability, offering an effective and robust solution for cross-instrument model adaptation.

### 5.5. Discussion

In this study, we observe significant differences in the performance of various model transfer methods across different parameters. Method 6, incorporating the BDA strategy, generally enhances cross-domain adaptation and prediction accuracy by aligning feature distributions. It shows the best performance in multiple component predictions of the pharmaceutical and corn datasets. Nevertheless, in specific cases like the corn oil content prediction, method 2 (fine-tuning all layers) achieves better results on a particular target instrument. This might be because method 2’s comprehensive parameter updating better adapts the model to the target domain’s features. In contrast, methods 3, 4, and 5, which only fine-tune certain layers, underperform in some component predictions, suggesting they may not fully utilize the target-domain data characteristics. These differences likely stem from variations in spectral features of different parameters and the model’s sensitivity to them. For some parameters with spectral features highly susceptible to instrument differences, more complex transfer methods are needed for distribution alignment. For others with relatively stable features, simple methods suffice.

This extended comparative experiment clearly establishes the methodological advantages of the proposed transfer learning approach, demonstrating superior performance not only over traditional calibration transfer methods (DS, PDS, SBC) but also over previously evaluated machine learning models (BDSER-InceptionNet, PLS, SVR, CNN). By consistently achieving higher R^2^ values and lower RMSE values across all predicted components, BDSER-InceptionNet provides a robust and effective framework for calibration transfer in spectral data analysis. These results validate the effectiveness of transfer learning in addressing the challenges posed by instrumental variations, representing a significant improvement over existing approaches and laying the foundation for more reliable and accurate predictions in practical spectroscopic applications.

In summary, the choice of model transfer method should consider the target-domain data characteristics and the differences between domains. While the BDA strategy usually improves transfer effectiveness, its performance is also influenced by data quantity, noise, and parameter settings. Future research will focus on optimizing the BDA strategy and exploring advanced model architectures to further enhance model generalization and prediction accuracy.

## 6. Conclusions

This study introduces a model transfer algorithm based on deep transfer learning, leveraging the BDSER-InceptionNet model and the Balanced Distribution Adaptation (BDA) strategy to effectively address the problem of insufficient model generalization caused by instrument differences in Near-Infrared (NIR) spectroscopy analysis. The BDSER-InceptionNet model integrates Depthwise Separable Convolution (DSC), multi-scale feature extraction (RX-Inception), and an attention mechanism (SE module), significantly enhancing feature extraction efficiency and model robustness. The BDA algorithm further optimizes the model’s cross-instrument adaptability by aligning the marginal and conditional distributions of the source and target domains. The superiority of this approach was validated through experiments on two publicly available datasets: tablet and corn. In the tablet dataset, the transfer strategy incorporating BDA achieved an R^2^ of 0.9860 in predictions on secondary instruments, with Root Mean Square Error (RMSE) and Mean Absolute Error (MAE) as low as 1.7489 and 1.4055, respectively, outperforming traditional methods such as Partial Least Squares (PLS), Support Vector Regression (SVR), and conventional Convolutional Neural Networks (CNNs). Corn dataset results: Predictions of moisture, oil, protein, and starch content demonstrated that the BDA-optimized model exhibited greater stability and prediction accuracy in multi-instrument scenarios. In the corn dataset, predictions of moisture, oil, protein, and starch content demonstrated that the BDA-optimized model exhibited greater stability and prediction accuracy in multi-instrument scenarios. Compared to traditional methods, this model’s reduced reliance on preprocessing and its efficient end-to-end analysis capabilities underscore its practical value. In comparative experiments with traditional transfer methods, conventional chemometric models were outperformed by the proposed algorithm, with results highlighting significant performance advantages. Notably, the method excelled in generalization across instruments, effectively mitigating spectral differences between devices and thereby enhancing transfer effectiveness and prediction accuracy. Looking ahead, this approach holds promise for broader application in NIR spectroscopy scenarios, offering reliable support for cross-instrument analysis and contributing to advancements in non-destructive testing technologies.

## Figures and Tables

**Table 1:** The hyperparameters in this study.

Dataset	Hyperparameters						
	Batch Size	LR ^1^	RX-Inception	Conv1	Dropout	F1 Linear	F2 Linear
Tablet	128	0.001	M1 ^2^ = 5,	In_c ^6^ = 1,	0.5	(100 × 25, 100)	(100, 1)
			M2 ^3^ = 9,	Out_c ^7^ = 16			
			M3 ^4^ = 15,				
			M4 ^5^ = 3				
Corn	8	0.001	M1 = 3,	In_c = 1,	0	(100 × 256, 100)	(100, 1)
			M2 = 5,	Out_c = 16			
			M3 = 13,				
			M4 = 3				

^1^ Learning Rate. ^2^ Kernel size of the convolutional layer in the first branch of the RX-Inception structure. ^3^ Kernel size of the convolutional layer in the second branch of the RX-Inception structure. ^4^ Kernel size of the convolutional layer in the third branch of the RX-Inception structure. ^5^ Kernel size of the convolutional layer in the fourth branch of the RX-Inception structure. ^6^ Number of input channels. ^7^ Number of output channels.

**Table 2:** Direct prediction results of the established models for A1 and A2.

Model	A1 ^1^			A2 ^2^		
	R^2^	RMSE	MAE	R^2^	RMSE	MAE
PLS	0.9671	2.6593	2.0511	−0.3750	17.1813	16.8522
SVR	0.9774	2.2040	1.8040	0.0542	14.2494	13.4485
CNN	0.9762	2.2000	1.7250	0.9606	2.8732	2.3384
Ours	0.9810	2.0243	1.6603	0.9812	2.0183	1.5892

^1^ Host instrument. ^2^ Secondary instrument.

**Table 3:** Prediction results of the six transfer methods on the pharmaceutical dataset.

Transfer Type	A1 ^1^ to A2 ^2^		
	R^2^	RMSE	MAE
Transfer1	0.9812	2.0183	1.5892
Transfer2	0.9856	1.7707	1.4206
Transfer3	0.9830	1.8629	1.4717
Transfer4	0.9815	1.9932	1.5702
Transfer5	0.9859	1.7710	1.4221
Transfer6	0.9860	1.7489	1.4055

^1^ Host instrument. ^2^ Secondary instrument.

**Table 4:** Direct prediction results of the moisture content model for m5, mp5, and mp6.

Content	Model	m5 ^1^		mp5 ^2^		mp6 ^3^	
		R^2^	RMSE	R^2^	RMSE	R^2^	RMSE
moisture	PLS	0.9771	0.0451	−26.6026	1.5652	−32.2404	1.7176
	SVR	0.9825	0.0394	−10.8817	1.0269	−12.5395	1.0962
	CNN	0.9666	0.0493	0.5535	0.2706	0.4773	0.3025
	Ours	0.9739	0.0473	0.5798	0.2509	0.5202	0.2805
oil	PLS	0.6856	0.1017	−2.6955	0.3485	−5.1764	0.4506
	SVR	0.8166	0.0776	0.6445	0.1081	0.3096	0.1507
	CNN	0.8863	0.0525	0.7650	0.0738	0.7943	0.0676
	Ours	0.8969	0.0488	0.7916	0.066	0.8445	0.0591
protein	PLS	0.7977	0.2278	0.4663	0.3698	−0.6115	0.6429
	SVR	0.9557	0.1066	−2.0602	0.8860	−2.8887	0.9987
	CNN	0.9621	0.0967	0.9264	0.1291	0.9474	0.1100
	Ours	0.9650	0.0926	0.9335	0.1246	0.9639	0.0942
starch	PLS	0.5685	0.4640	−1.7222	1.1652	−4.1174	1.5978
	SVR	0.8793	0.2453	−4.1623	1.6047	−2.0259	1.2285
	CNN	0.9403	0.1668	0.8387	0.2695	0.8820	0.2248
	Ours	0.9474	0.1574	0.8461	0.253	0.8642	0.2384

^1^ Host instrument. ^2^ Secondary instrument. ^3^ Secondary instrument.

**Table 5:** The prediction results for the four components of the maize dataset after applying various transfer learning methods are shown below.

Content	Transfer Type	m5 ^1^ to mp5 ^2^		m5 to mp6 ^3^	
		R^2^	RMSE	R^2^	RMSE
moisture	Transfer1	0.5798	0.2509	0.8445	0.0591
	Transfer2	0.8335	0.1337	0.9159	0.0493
	Transfer3	0.8430	0.1293	0.7943	0.0676
	Transfer4	0.7684	0.1482	0.6917	0.1682
	Transfer5	0.8533	0.1247	0.7960	0.1465
	Transfer6	0.8589	0.1245	0.8226	0.1393
oil	Transfer1	0.7916	0.0660	0.8445	0.0591
	Transfer2	0.7760	0.0721	0.9159	0.0493
	Transfer3	0.8479	0.0670	0.9105	0.0545
	Transfer4	0.7742	0.0701	0.7581	0.0674
	Transfer5	0.8280	0.0690	0.8930	0.0585
	Transfer6	0.8482	0.0636	0.9042	0.0531
protein	Transfer1	0.9335	0.1246	0.9639	0.0943
	Transfer2	0.9452	0.1100	0.9327	0.1186
	Transfer3	0.9671	0.0893	0.9490	0.1200
	Transfer4	0.9164	0.1309	0.9455	0.1075
	Transfer5	0.9617	0.0894	0.9497	0.1017
	Transfer6	0.9688	0.0859	0.9572	0.0985
starch	Transfer1	0.8461	0.2530	0.8642	0.2384
	Transfer2	0.8696	0.2390	0.9464	0.1523
	Transfer3	0.8919	0.2138	0.9544	0.1567
	Transfer4	0.8678	0.2476	0.9163	0.1591
	Transfer5	0.9120	0.2161	0.9617	0.1377
	Transfer6	0.9141	0.2123	0.9554	0.1478

^1^ Host instrument. ^2^ Secondary instrument. ^3^ Secondary instrument.

**Table 6:** Coefficient of determination ($R^{2}$) using different transfer algorithms on pharmaceutical and corn datasets.

Predicted Component	The $R^{2}$ of the Model Transferred from the Primary Instrument to the Secondary Instrument			
	DS ^1^	PDS ^2^	SBC ^3^	Ours
Tablet: A2	0.8117	0.9556	0.8942	0.9860
Moisture: mp5	0.8641	−0.2519	0.2307	0.8589
Moisture: mp6	0.8515	−0.9423	0.3516	0.8226
Oil: mp5	0.7253	0.0212	0.6904	0.8482
Oil: mp6	0.7246	0.1558	0.7285	0.9042
Protein: mp5	0.8889	0.7035	0.8292	0.9688
Protein: mp6	0.8561	0.1226	0.9014	0.9572
Starch: mp5	0.3713	−0.3273	0.5330	0.9141
Starch: mp6	0.7439	−7.9500	0.7552	0.9554

^1^ Direct standardization. ^2^ Piecewise direct standardization. ^3^ Slope and bias correction.

**Table 7:** Root mean square error (RMSE) using different transfer algorithms on pharmaceutical and corn datasets.

Predicted Component	RMSE of the Model Transferred from the Primary Instrument to the Secondary Instrument			
	DS ^1^	PDS ^2^	SBC ^3^	Ours
Tablet: A2	6.3586	3.0885	4.7653	1.7489
Moisture: mp5	0.1098	0.3333	0.2613	0.1245
Moisture: mp6	0.1148	0.4152	0.2399	0.1393
Oil: mp5	0.0950	0.1794	0.1009	0.0636
Oil: mp6	0.0951	0.1666	0.0945	0.0531
Protein: mp5	0.1688	0.2758	0.2093	0.0858
Protein: mp6	0.1921	0.4744	0.1591	0.0985
Starch: mp5	0.5600	0.8137	0.4826	0.2123
Starch: mp6	0.3574	2.1129	0.3495	0.1478

^1^ Direct standardization. ^2^ Piecewise direct standardization. ^3^ Slope and bias correction.

## Data Availability

Publicly available datasets: Tablet: https://eigenvector.com/resources/data-sets/#idrc2002 (accessed on 10 December 2024); Corn: https://eigenvector.com/resources/data-sets/#corn-sec (accessed on 10 December 2024).
